# Factors Associated with Clostridioides (Clostridium) Difficile Infection and Colonization: Ongoing Prospective Cohort Study in a French University Hospital

**DOI:** 10.3390/ijerph18147528

**Published:** 2021-07-15

**Authors:** Nagham Khanafer, Philippe Vanhems, Sabrina Bennia, Géraldine Martin-Gaujard, Laurent Juillard, Thomas Rimmelé, Laurent Argaud, Olivier Martin, Laetitia Huriaux, Guillaume Marcotte, Romain Hernu, Bernard Floccard, Pierre Cassier, Study Group

**Affiliations:** 1 International Center for Infectiology Research (CIRI), Inserm U1111, CNRS UMR5308, ENS de Lyon, Lyon 1 University, 69342 Lyon, France; philippe.vanhems@chu-lyon.fr; 2 Department of Hygiene, Epidemiology, and Prevention, Edouard Herriot Hospital, Hospices Civils de Lyon, 69437 Lyon, France; sabrina.bennia@chu-lyon.fr; 3 European Study Group for Clostridioides Difficile (ESGCD), 4001 Basel, Switzerland; 4 INSERM, F-CRIN, Réseau Innovative Clinical Research in Vaccinology (I-REIVAC), 75679 Paris, France; 5 Geriatric Department, Edouard Herriot Hospital, Hospices Civils de Lyon, 69437 Lyon, France; geraldine.martin-gaujard@chu-lyon.fr; 6 Nephrology Department, Edouard Herriot Hospital, Hospices Civils de Lyon, 69002 Lyon, France; laurent.julliard@chu-lyon.fr; 7 Anesthesia and Intensive Care Unit, Edouard Herriot Hospital, Hospices Civils de Lyon, 69437 Lyon, France; thomas.rimmele@chu-lyon.fr (T.R.); olivier.martin@chu-lyon.fr (O.M.); laetitia.huriaux@chu-lyon.fr (L.H.); guillaume.marcotte@chu-lyon.fr (G.M.); bernard.floccard@chu-lyon.fr (B.F.); 8 EA 7426 PI3 (Pathophysiology of Injury-Induced Immunosuppression), Lyon 1 University, Hospices Civils de Lyon, Biomérieux, 69437 Lyon, France; 9 Medical Intensive Care Unit, Edouard Herriot Hospital, Hospices Civils de Lyon, 69437 Lyon, France; laurent.argaud@chu-lyon.fr (L.A.); romain.hernu@chu-lyon.fr (R.H.); 10 Environnemental Laboratory, Institut des Agents Infectieux, Hospices Civils de Lyon, 69317 Lyon, France; pierre.cassier@chu-lyon.fr; 11 Edouard Herriot Hospital, Hospices Civils de Lyon, 69437 Lyon, France; study.group@chu-lyon.fr

**Keywords:** asymptomatic colonization, *Clostridioides difficile*, follow-up, hospital, risk factors

## Abstract

Introduction: *Clostridioides (Clostridium) difficile* can be isolated from stool in 3% of healthy adults and in at least 10% of asymptomatic hospitalized patients. *C. difficile*, the most common cause of hospital-acquired infectious diarrhea in the developed world, has re-emerged in recent years with increasing incidence and severity. In an effort to reduce the spread of the pathogen, published recommendations suggest isolation and contact precautions for patients suffering from *C. difficile* infection (CDI). However, asymptomatic colonized patients are not targeted by infection control policies, and active surveillance for colonization is not routinely performed. Moreover, given the current changes in the epidemiology of CDI, particularly the emergence of new virulent strains either in the hospital or community settings, there is a need for identification of factors associated with colonization by *C. difficile* and CDI. Methods and analysis: We are carrying out a prospective, observational, cohort study in Edouard Herriot Hospital, Hospices Civils de Lyon, a 900-bed university hospital in Lyon, France. All consecutive adult patients admitted on selected units are eligible to participate in the study. Stool samples or rectal swabs for *C. difficile* testing are obtained on admission, every 3–5 days during hospitalization, at the onset of diarrhea (if applicable), and at discharge. Descriptive and logistic regression analyses will be completed to mainly estimate the proportion of asymptomatic colonization at admission, and to evaluate differences between factors associated with colonization and those related to CDI. Ethics: The study is conducted in accordance with the ethical principles of the Declaration of Helsinki, French law, and the Good Clinical Practice guidelines. The study protocol design was approved by the participating units, the ethics committee and the hospital institutional review board (Comité de protection des personnes et Comission Nationale de l’Informatique et des Libertés; N°: 00009118). Dissemination: The results of this study will be disseminated by presenting the findings locally at each participating ward, as well as national and international scientific meetings. Findings will be shared with interested national societies crafting guidelines in CDI.

## 1. Introduction

*C. difficile* can be isolated from stool in 3% of healthy adults and in at least 10% of asymptomatic hospitalized patients. It is widely distributed in soil and in the intestinal tracts of animals [1]. *C. difficile*, the most common cause of hospital-acquired infectious diarrhea in the developed world, has re-emerged in recent years with increasing incidence and severity [2]. The clinical spectrum of *C. difficile* infection (CDI) varies in severity from asymptomatic carriage and self-limited mild, watery diarrhea, to pseudomembranous colitis (PMC), intestinal perforation, toxic megacolon, sepsis, fulminant colitis, and death [3]. Variability in host factors may explain the wide spectrum of symptoms and the disease’s course [4]. Over the past decade, a highly virulent *C. difficile* strain, commonly known as 027/NAP1/BI, has emerged worldwide and has been implicated in large hospital outbreaks with increased severity, frequent recurrence, and significant mortality [5,6,7,8,9,10,11]. Its impact in healthcare settings is considerable, in terms of morbidity, mortality and financial cost [12]. 

Antibiotic use, advanced age, increased severity of underlying illness, prior hospitalization, use of feeding tubes, gastrointestinal surgery, and use of proton-pump inhibitors (PPI) are the major risk factors associated with CDI [4]. Transmission of *C. difficile* occurs by way of the fecal–oral route after transient contamination of healthcare workers (HCW), patients and the environment. As an obligate anaerobic bacterium, vegetative *C. difficile* cells die within minutes after exposure to air. On the contrary, the organism produces spores, the transmissible form of *C. difficile*, which are extremely resistant to most disinfectants and can survive up to several months in a hospital room after a patient with CDI has been discharged [13,14,15,16]. In an effort to reduce the spread of the pathogen, published recommendations suggest isolation and contact precautions for patients suffering from CDI. However, asymptomatic colonized patients are not targeted by infection control policies, and active surveillance for colonization is not routinely carried out [17]. In comparison to patients not colonized with *C. difficile*, asymptomatic colonized patients were reported as having a lower risk of CDI [18]. Nevertheless, a systematic review and meta-analysis showed that colonized patients have an almost six times higher risk of developing an infection compared with non-colonized patients. Ignoring the toxinogenicity of colonizing strains in previous studies can explain the protective role of carriage [17]. Moreover, given the current changes in the epidemiology of CDI, particularly the emergence of new virulent strains (027, 078, 012 244, …) [19,20,21,22,23] either in the hospital or community settings, there is a need for identification of factors associated with colonization by *C. difficile* and CDI. The objectives of this prospective cohort study are the following:

− To estimate the proportion of asymptomatic colonization at admission.
− To describe the association between colonization with either toxigenic or non-toxigenic strains of *C. difficile* and subsequent infection.
− To calculate the delay between acquisition of *C. difficile* and the onset of symptoms related to CDI.
− To identify the factors associated with colonization and infection.

## 2. Methods and Analysis

### 2.1. Study Design, Location and Patients

A prospective, observational, cohort study was undertaken in Edouard Herriot Hospital, Hospices Civils de Lyon, a 900-bed university hospital in Lyon, France. The diagram below summarizes the study design.

All consecutive patients 18 years of age or older admitted on selected units were eligible to participate in the study. Outpatients, day-care patients, patients with hemodynamic instability, patients who received palliative care, or patients who refused to participate or who are unable to participate in the informed consent process on their own behalf or represented by a surrogate were excluded. Patients with diarrhea suspected to be related to *C. difficile* had their microbiological workup carried out following the management of the physician in charge. 

### 2.2. Sample Size

No published French data were available to contribute to the estimation of the needed sample size. Therefore, a convenience, non-probabilistic, consecutive sample, limited in time, was recruited. Moreover, as the main goal of the study was primarily descriptive (prevalence of colonization at admission and at different follow-up times), a sample sizing was not required. 

### 2.3. Expected Study Period

From March 2017 to December 2020.

### 2.4. Participating Wards

The selected units were those with a historically high incidence of CDI. According to our surveillance study conducted since 2006, we decided to include patients admitted to: the intensive care unit (ICU, 3 units), geriatrics (3 units) and nephrology (one unit). 

### 2.5. Ethics and Dissemination

The study was conducted in accordance with the ethical principles of the Declaration of Helsinki, French law, and the Good Clinical Practice guidelines. The regulatory framework of this study was defined by Jardé’s law in accordance with the European General Regulation of Data. Thus, the study protocol’s design was approved by the participating units, the ethics committee and the hospital institutional review board (Comité de protection des personnes et Comission Nationale de l’Informatique et des Libertés; N°: 00009118). There were no safety concerns for enrolled patients. According to French law, informed consent for this observational, non-interventional study is not obligatory. However, for all admitted patients and/or their family and/or their reliable person, detailed oral and written information in French was delivered. Participants were informed that their involvement was voluntary and that they had the right to withdraw at any time from the study. To ensure anonymity, no personal identifying details were kept with either the samples or the collected data. Moreover, a poster for information was displayed in each participating ward. During the course of the study, the information collected was not disclosed to anyone other than the study personnel. At the conclusion of the study, only study staff would have full access to the final dataset.

The results of this study will be disseminated by presenting the findings locally at each participating wards, as well as national and international scientific meetings. Findings will be shared with interested national societies crafting guidelines in CDI. We planned to publish the results in peer-reviewed journals.

### 2.6. Patient and Public Involvement

Patients were not involved in the design or implementation of this study. Study participants gave their consent and will be given access to the final publication of the study results upon request.

### 2.7. Definitions

CDI is defined as follows: the presence of diarrhea and a positive *C. difficile* toxins test. Diarrhea is defined as three loose stools within at least one 24 h period.

CDI is considered to be health care-associated if symptoms began 48 h or more after admission, if CDI is diagnosed within 4 weeks after discharge from any health care institution. Recurrence is defined as a second episode of CDI within 60 days after the first episode. Asymptomatic *C. difficile* colonization is defined by the detection of *C. difficile* in the absence of symptoms of infection. Colonization is considered as health care-associated if the patient is not colonized at inclusion with a positive test without diarrhea at least 48 h later.

### 2.8. Clinical Data

Data on demographic information, known risk factors, and potential confounding factors were collected. In particular, information about the use of various medications during the 60 days before, as well as during hospitalization was compiled for all patients. The specific start and stop dates of antibiotics were also recorded. Patients were followed daily until ward discharge, death, or withdrawal from the study. Patients were contacted three times (by day 30, day 60 and day 90) after inclusion to determine whether diarrhea had occurred in the interim/interval. Outcomes studied are recurrence of CDI, death, and related complications (megacolon, colectomy, PMC, septic shock, admission to ICU, and readmission for CDI). For any death, clinicians in charge of patients will judge independently whether CDI was a primary cause, a contributory cause, or unrelated to the cause of death.

### 2.9. Clinical Samples

**Figure 1:**
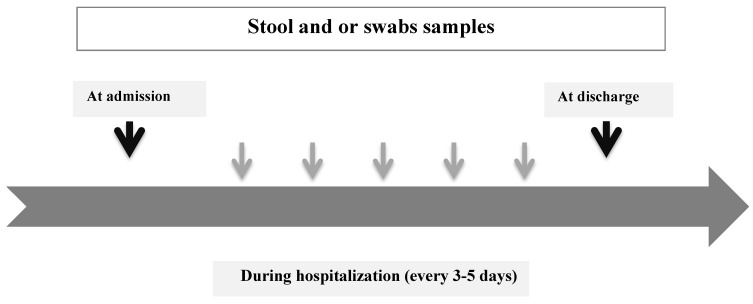
Clinical samples during ward stay.

HCW were asked to collect stool samples for *C. difficile* testing (Figure 1) on admission, every 3–5 days and at discharge. Whenever a stool sample was impossible to collect, a rectal swab was performed. An appropriate container was delivered to each participating ward to collect samples, which were transported daily to the laboratory to be analyzed.

### 2.10. Laboratory Assays

Diagnosis of CDI was based on current recommendations, using a two-step algorithm with a combined test of GDH and toxins (C. DIFF QUIK CHEK COMPLETE^®^, Alere, Waltham, MA, USA) followed by PCR (GeneXpert^®^ Systems, Cepheid, Sunnyvale, CA, USA) in cases of non-contributing result. 

For a GDH-positive sample, quantitative culture was performed. At least 3 colonies by culture were characterized at the National Reference Center (NRC) by PCR multiplex detection of tcdA, tcdB, tpi, tcdC, cdtA, cdtB genes and PCR ribotyping [24,25].

### 2.11. Statistical Analysis

Epidemiological and molecular data were collected and interpreted independently. Participants were categorized into different groups, according to status with respect to CDI, colonization and origin of acquisition (acquired in our hospital, imported from another hospital or community acquired). 

Statistical analyses were performed with the Statistical Package for Social Sciences (SPSS, version 19.0 for Windows, SPSS, Inc., Chicago, IL, USA) and R statistical software (R Foundation for Statistical Computing, Vienna, Austria. URL: http://www.R-project.org/ accessed on 14 July 2021, Version 2.14.2).

### 2.12. Data Entry

Location of and information on sampling wards and data were recorded on specific paper clinical record folders (CRFs) and an electronic database. Epi info was used to create a digital survey and data entry. Different tables with data at admission, during hospital stay and follow-up, and biological and microbiological data were created. For each table, a unique key was used to link it with the main questionnaire. 

### 2.13. Descriptive Analysis

The following characteristics of included patients were described: age, gender, diagnosis on admission, co-morbidities, nutritional status, diarrheal symptoms and their duration, prior CDI history, results of microbiological and biological tests, and data on follow-up. Biological features: leukocytes count, neutrophils, C-reactive protein, serum albumin, creatinine and lactate dehydrogenase values were collected on days −1 to +1 relative to the day of each stool sample. Data on exposure to risk factors associated with CDI were also compiled—these included previous hospitalization, nursing home residency, antibiotics (ATB), laxative use, antisecretory drugs, and surgical procedures (endoscopy, percutaneous gastrostomy, nasogastric feeding, gastrointestinal surgery, and parenteral nutrition). Data on hospital stay were described as follows: source (or origin) and length of stay (LOS at ward of inclusion and at hospital). 

Antibiotics were grouped into the following classes: first, second, third and fourth generation cephalosporins, fluoroquinolones, penicillins and b-lactamase inhibitor combinations, other penicillins, carbapenems, glycopeptides, macrolides, aminoglycosides, metronidazole, and other ATB. 

Summary tables (descriptive statistics and/or frequency tables) and/or figures are provided for all baseline variables as appropriate. Continuous variables were summarized with descriptive statistics (n, mean, range, median and interquartile range). Frequency counts and percentage of subjects within each category were provided for categorical data. Student’s or the Mann–Whitney U tests were used for comparison of two groups, and one-way analysis of variance with Mann–Whitney rank sum test and Dunn’s test as a post hoc test for comparison of more than two groups. The Chi-square or Fisher’s exact tests were used to compare categorical variables. Two-tailed *p* < 0.05 values were considered statistically significant in all tests.

For cases with isolation of *C. difficile*, data on strain typing were detailed: resistance to ATB, ribotypes and the presence of binary toxins. 

### 2.14. Rates of Colonization, CDI and Mortality

Colonization and CDI rates (i.e., cases per 10,000 patient-days or per 1000 admissions as appropriate) during the study period were calculated according to different categories: patients in specific ward, origin of acquisition, etc. Hospital-specific incidence was calculated using the denominators obtained from individual hospitals for the total number of patients discharged from participating wards and patient-days during the study period. The specific rate by ward was calculated as the number of cases diagnosed at this ward during the study period divided by the total number of ward admissions in the study period. A Poisson regression model was carried out to assess longitudinal trend. Model fit was assessed through Akaike’s Information Criterion, deviance and Pearson chi-square statistics. Mortality rate in included patients was calculated as the sum of patients who died within 90 days of follow up after inclusion per total number of patients included in the analysis. This rate will be computed for CDI confirmed cases (global rate and mortality considered by clinicians as related to CDI), colonization and patients not colonized or infected by *C. difficile*. 

### 2.15. Estimation of Factors Associated with Colonization and/or CDI

Univariate analysis was performed (crude (i.e., unadjusted)) to evaluate the association between exposures and outcome of interest (i.e., colonization and confirmed CDI). To ensure that case patients and control patients had similar risks of exposure to *C. difficile*, a frequency matching approach was used that linked all affected patients and controls within each stratum defined by a combination of values for hospital and length of stay. The period of risk is defined as the time from admission until diagnosis of *C. difficile* infection or colonization (for infected and colonized patients, respectively) or discharge (for controls).

Variables were retained in the multivariate regression model at the 0.2 level in univariate analyses and those we judged a priori to be clinically sound. 

In this case, different independent covariates (exposures and/or confounders) were introduced in the logistic model if each of these covariates was linearly related to the logit of the outcome. An examination of residuals, leverage, and influence statistics was carried out to assess model adequacy to ensure that valid inferences could be made from the estimated beta coefficients. The Hosmer–Lemeshow goodness of fit test was carried out to assess the model–data fit. The number of covariates in the model was considered. A full model was defined as being free from overfitting and less likely to have multicollinearity issues. The procedure to reduce the number of covariates involves a strategy for comparing relevant models, based either on testing significance of the covariates, on a comparison of estimates of the error variances, or on a comparison of the changes of the beta coefficient between the model with and without the covariates under assessment. At each step, the variable showing the smallest contribution to the model was removed to obtain a model that only contained the covariates that provided important information about the outcome. The measure of the association was presented by the odds ratio (OR) with a 95% confidence interval (95% CI). 

### 2.16. Description of Prognosis of Included Patients and Survival Analyses 

The outcomes of colonized patients, CDI-confirmed patients and patients who had neither CDI nor colonization were fully described: good without complications, proportions of complications, relapses, in-hospital death, and death at day 30, day 60 and day 90. Patient outcomes were analyzed until in-patient death or last contact at the end of follow up. The survival time was computed as the interval between the date of inclusion and either the occurrence of the outcome (death) or censoring. A sub-analysis in patients with CDI, PCR-ribotype, and tcdC Δ117 deletion status, and the presence or absence of binary toxin were used as the genomic covariates.

The distribution of continuous variables was checked. Chi-square or Fisher’s exact test (for qualitative variables) and the Wilcoxon or Mann–Whitney U test (for quantitative variables) were used to evaluate differences between patient subgroups. 

To test for differences between subgroups and to estimate survival probability, we carried out Kaplan–Meier analysis with the log rank test. Univariate and multivariate (Cox regression model and/or competitive risks) analyses were performed to identify factors associated with poor prognosis.

## 3. Discussion

A better understanding of the role of asymptomatic carriage in CDI transmission, and the available measures to reduce that risk (e.g., duration of shedding, contagiousness, infectious dose), may be essential to guide for better preventive and therapeutic approaches, including the appropriate use of antibiotics and how these colonized patients impact transmission within healthcare settings [26].

Asymptomatic carriage of *C. difficile* at admission is associated with a significant risk of progression to symptomatic CDI. Consequently, the screening of asymptomatic carriers may represent a good opportunity to reduce the incidence of CDI [27]. However, information on the factors associated with disease onset is limited. A recent Canadian study identified several factors that are associated with CDI among colonized patients. Whether modifying these variables could decrease the risk of CDI needs to be investigated [28]. 

An investigation to compare the burden of environmental shedding of toxigenic *C. difficile* among asymptomatic carriers, CDI patients and non-carriers in non-epidemic settings showed more than residual contamination in 41% of carrier rooms—24% of these were heavily contaminated. Moreover, multivariate analysis showed that the contamination score of rooms of asymptomatic carriers did not differ from rooms of CDI patients [29]. 

To the best of our knowledge, this study is the first French project on assessing longitudinal colonization by *C. difficile*. It addresses several questions such as the estimation of the prevalence of asymptomatic colonization at admission, the incidence of hospital acquisition and the delay between acquisition and the onset of symptoms related to CDI and if there is a difference between factors associated with colonization and infection. In addition, we decided to follow included patients for 90 days after inclusion to quantify unfavorable outcomes. Most studies of patients with CDI have focused mainly on outcomes such as mortality, length of stay, recurrence, and cost. In addition to all of these, we still need more data on complications; this will provide practitioners with a good summary of the clinical profile of the burden of this infection. Moreover, the typing of multiple colonies in colonized patients could help us to evaluate the association between colonization with either toxigenic or non-toxigenic strains of *C. difficile* and subsequent CDI. Finally, the typing of many colonies will allow us to investigate the possibility of being colonized by different PCR-ribotypes.

This study was not conducted to assess its feasibility, but some questions were asked to identify barriers and facilitators of implementation before the latter was carried out: What will be the acceptancy rate of patients? What are the risks and benefits? How can HCW be convinced to carry out sampling? What are the costs and impacts of the current situation?

Numerous interventions involving different steps and approaches should be achieved before implementation, such as (1) the people involved and their roles; (2) information exchange; (3) how to communicate with HCW; (4) clear explanation of objectives and risks/benefits for patients and HCW; (5) implementation and evaluation, etc.

Important barriers for HCW included time constraints and lack of applicability due to patients’ characteristics. Important facilitators included provider motivation to screen patients at high risk of colonization at admission, its positive impact on patient outcomes and its positive impact through limiting the consequences of isolation for patients and HCW. 

## 4. Strengths and Limitations

The motivation in designing a prospective cohort including all consecutive adults admitted to the participating wards is to assess the colonization of admission and during the hospital stay. In addition, all participating patients were prospectively followed for 3 months after inclusion, which has not been achieved in previous studies evaluating asymptomatic colonization by *C. difficile*. 

We acknowledge some limitations. First, it is a single-center study but the expected high number of samples to be collected in this study will allow for powerful statistical analysis. Second, colonization after discharge from the participating ward cannot be assessed. Finally, the environmental contamination in participating wards cannot be assessed for logistic reasons, which do not permit us to estimate the shedding of *C. difficile* in the rooms occupied by colonized and/or infected patients in comparison to other patients.

## 5. Conclusions

This study will provide important baseline data from a large French university hospital on the colonization rates of *C. difficile*. It is known that individuals colonized by *C. difficile* may acquire some protection from progression to disease. Yet, they also have the potential to contribute to transmission in healthcare settings—the identification of factors associated with colonization by *C. difficile* and CDI are of a great interest.

## Data Availability

The data that support the findings of this study are not publicly available due to ongoing analyses.

## Appendix A

Study group (Edouard Herriot Hospital): Valérie Cerro, Dubreuil Justine, Josiane Bourgeay, Sylvie Pereira, Agnes Cleux, Caroline Deneriaz, Christain Labro and Sylvie-de-la- Salle.
